# REMOTE-ILD study: Description of the protocol for a multicentre, 12-month randomised controlled trial to assess the clinical and cost-effectiveness of remote monitoring of spirometry and pulse oximetry in patients with interstitial lung disease

**DOI:** 10.1136/bmjresp-2023-002067

**Published:** 2024-02-28

**Authors:** Sarah Barth, Colin Edwards, Rebecca Borton, Dan Beever, Wendy Adams, Gisli Jenkins, Elena Pizzo, Iain Stewart, Melissa Wickremasinghe

**Affiliations:** 1 Respiratory Medicine, Imperial College Healthcare NHS Trust, London, UK; 2 National Heart and Lung Institute, Imperial College London, London, UK; 3 patientMpower Ltd, Dublin, Ireland; 4 Action For Pulmonary Fibrosis, Peterborough, UK; 5 University College London, London, UK

**Keywords:** Interstitial Fibrosis, Respiratory Function Test

## Abstract

**Introduction:**

Remote monitoring of home physiological measurements has been proposed as a solution to support patients with chronic diseases as well as facilitating virtual consultations and pandemic preparedness for the future. Daily home spirometry and pulse oximetry have been demonstrated to be safe and acceptable to patients with interstitial lung disease (ILD) but there is currently limited evidence to support its integration into clinical practice.

**Aim:**

Our aim is to understand the clinical utility of frequent remote physiological measurements in ILD and the impact of integrating these into clinical practice from a patient, clinical and health economic perspective.

**Methods and analysis:**

132 patients with fibrotic ILD will be recruited and randomised to receive either usual care with remote digital monitoring of home spirometry and pulse oximetry or usual care alone for 12 months. All participants will complete health-related quality of life and experience questionnaires.

The primary outcome compares the availability of spirometry measurements within the 2 weeks preceding planned clinic appointments. Secondary outcomes will explore other aspects of clinical and cost-effectiveness of the remote monitoring programme.

**Ethics and dissemination:**

The study has been approved by the Camden and Kings Cross Research Ethics Committee (22/LO/0309). All participants will provide informed consent.

This study is registered with www.clinicaltrials.gov (NCT05662124).

The results of the study will be submitted for presentation at regional and national conferences and submitted for peer-reviewed publication. Reports will be prepared for study participants with the support from our public involvement representatives through the charity Action for Pulmonary Fibrosis.

**WHAT IS ALREADY KNOWN ON THIS TOPIC**

- Regular home spirometry and pulse oximetry are safe and feasible for patients with interstitial lung disease (ILD). However, its utility in supporting clinical decision-making in clinical practice is currently unclear.

**WHAT THIS STUDY ADDS**

- This is the first randomised controlled trial of remote monitoring versus usual clinical care in ILD, which aims to establish if there are differences in clinical care and health economic outcomes over a 12-month period.

**HOW THIS STUDY MIGHT AFFECT RESEARCH, PRACTICE OR POLICY**

- This study aims to understand whether there is a useful role for remote monitoring in clinical care of patients with ILD and the resource implications required for its incorporation into clinical care.

## Introduction

Interstitial lung disease (ILD) is an umbrella term describing a spectrum of diseases of progressive scarring of the lungs causing progressive breathlessness.^1^ In some cases, for example, in association with autoimmune disease, fibrosis develops following an inflammatory process in the lung parenchyma. In others, fibrosis develops without pre-existing inflammation. The most common form of ILD is idiopathic pulmonary fibrosis (IPF), which has a reported mean incidence of 0.12 cases/10000^2^ and is rising by up to 5% per year.^3^ Treatment of ILD aims to reduce any inflammation to prevent development to fibrosis, reduce development of further fibrosis, reduce lung inflammation and treat symptoms.

Current guidelines for the management of IPF^4 5^ recommend that patients are reviewed by a respiratory specialist every 4–6 months with lung function testing beforehand to monitor progression of disease and response to their current therapeutic regime. There is no formal guidance for the monitoring of patients with other forms of ILD but in the UK most patients are seen approximately 6 monthly with lung function testing.^6^ Lung function monitoring in ILD usually includes assessment of oxygen saturations, spirometry and gas transfer measurements.

Remote monitoring of physiological measurements has been proposed as an additional approach to monitoring chronic diseases. Benefits of remote spirometry and oximetry for patients with ILD have been suggested to include earlier detection of deterioration^7^ or acute illness and personalisation of care,^8^ reduced travel to hospitals,^9^ support of virtual consultations, and increased patient awareness and engagement in management of their lung disease which may support better psychological health.^10–12^

Previous studies have demonstrated that patients with ILD are willing and able to measure their spirometry at home frequently^13–17^ for up to 12 months.^18 19^ These studies have demonstrated that home handheld spirometry correlates well with lab-based spirometry,^7^ although home spirometry often underestimates values compared with lab-based spirometry. Currently, there is no evidence for optimal frequency of spirometry measurements in ILD and no guidelines for spirometry performed outside of a formal lung function laboratory.^20^ A previous randomised controlled trial of remote spirometry uploaded to a bespoke platform via Bluetooth did not meet its primary endpoint of improved health-related quality of life (HRQoL) assessed using the Kings Brief Interstitial Lung Disease Questionnaire but did demonstrate improvements in the psychological domains in those with home monitoring compared with those who did not.^10^

Oxygen saturation is a key physiological measurement of severity of lung disease. Remote pulse oximetry was implemented extensively during the COVID-19 pandemic to manage patients in the community and reduce hospital admissions and appeared to be safe but of limited impact.^21 22^ Preliminary studies by our group have shown that daily home pulse oximetry in patients with ILD for clinician review is feasible over a 3-month period with 83% of participants providing measurements on at least 70% of study days, and 72% of participants providing measurements at least three times/week.^23^

There are limited data regarding the use of remote pulse oximetry for monitoring ILD. It has been demonstrated that short-term continuous oximetry could be used to determine the need for ambulatory oxygen therapy but the economic implications of this were not measured.^24^ Previous studies show that patients with chronic lung diseases, including ILD, found knowing their oxygen saturations reassuring and helped guide their activity levels and oxygen consumption.^25^ Furthermore, clinicians have identified pulse oximetry as the variable that they think would be most beneficial for home monitoring in ILD.^26^

Currently remote monitoring in ILD is not widely used in clinical practice.^27^ Common barriers cited are organisational structure and lack of resources, as well as lack of evidence for efficiency and technical issues.^26^ Other concerns raised about integrating new digital health systems include both data overload, but also that artificial intelligence may not be able to detect subtler nuances, such as missing data may represent periods of either good health or deterioration.^28^

Our randomised controlled study aims to explore whether there is a difference in health and economic outcomes by comparing a cohort of patients who have been asked to monitor their pulse oximetry and spirometry with regular clinical review in addition to their usual clinical care compared with a cohort who are receiving usual clinical care alone.

## Methods and analysis

REMOTE-ILD is a prospective multicentre randomised controlled interventional and observational trial comparing the addition of remote monitoring of home spirometry and oximetry to usual care alone.

### Objectives

The objective of the study is to compare the clinical and cost-effectiveness of remote digital monitoring of home spirometry and pulse oximetry with usual care in the clinical management of patients with ILD.

#### Selection of participants

One hundred and thirty-two participants with fibrotic ILD will be recruited from ILD clinics at participating sites across the UK. Participants must have a diagnosis of fibrotic ILD as confirmed by their local multidisciplinary team meeting with radiological evidence of parenchymal lung fibrosis of any aetiology or radiological or pathological subtype.

A list of recruiting sites is available on www.clinicaltrials.gov and on request from the corresponding author.

Eligible patients who meet the entry criteria will be invited to consent. Most participants will be identified through outpatient clinics but recruitment will not be restricted to this route.

#### Inclusion criteria

- Diagnosis of fibrotic ILD as agreed by a specialist ILD multidisciplinary team.
- Age ≥18 years.
- Owns a smartphone or electronic tablet device which they are able to use either independently or with support from a carer or family member.
- Has a mobile telephone number, email address and access to the internet at home.
- In clinic lung function assessment within the last 6 months prior to study entry including spirometry and gas transfer measurement.
- Intention for at least two outpatient reviews during the observation period.
- Willing to allow home monitoring of their health including spirometry and pulse oximetry data.
- Understands how to use mobile technology (eg, Has downloaded and used other ‘apps’ on their mobile device; uses email).
- Demonstrates willingness to measure spirometry and pulse oximetry three time weekly for the duration of the observation period.
- Fluent in English.
- Written or electronic informed consent.

#### Exclusion criteria

- Unable to fulfil all inclusion criteria.
- Cognitive impairment (which would limit participant’s ability to understand the study or the procedures involved).
- History of difficulty performing spirometry at previous clinic testing.
- Contraindications to spirometry (eg, previous pneumothorax, unstable cardiac status, known aortic or cerebral aneurysm).
- Serious concomitant conditions which place the patient at high risk of respiratory distress.
- Recent (within the last 6 weeks) or current participation in another interventional clinical research project.

### Study design

The study design is an unblinded randomised control study with parallel arms where participants will be randomly allocated (1:1) to either the remote monitoring arm or usual care arm.

Following informed consent, baseline clinical data collected as part of usual clinical National Health Service (NHS) care will be used for the purpose of the study including previous medical history, all medication usage, previous lung function tests and radiology results. All participants will be asked to complete HRQoL questionnaires (modified Medical Research Council (mMRC) dyspnoea scale, K-BILD Questionnaire, EuroQol 5-Dimension 5-Level (EQ-5D-5L) questionnaire, Generalised Anxiety Disorder-7 (GAD-7) questionnaire) and Patient Activation Measure (PAM) at baseline.

Participants will then be randomly allocated to either the remote monitoring arm or usual care arms.

Remote monitoring arm:

Participants who are allocated to the active remote monitoring arm will be provided with a portable handheld spirometer (Spirobank Smart, MIR, www.spirometry.com) and pulse oximeter (model 3230, Nonin; www.nonin.com) linked to an electronic health application (patientMpower, www.patientMpower.com). Participants will be trained to undertake remote spirometry and pulse oximetry and they will be asked to perform measurements three times per week including two spirometry blows and one resting pulse oximetry reading on each occasion. All readings will automatically be captured via the patientMpower electronic health application and will be immediately visible to their clinical care team via a secure web-based portal. All spirometry attempts will be analysed in real time using artificial intelligence software (ArtiQ, www.artiq.eu)^29^ to assess the quality of the spirometry according to European Respiratory Society (ERS)/American Thoracic Society (ATS) criteria^20^ provided with real time feedback given to participants. Participants will be reminded to record their measurements once weekly.

Participating centres will be asked to monitor these remotely recorded measurements at least every 2 weeks and at, or before, planned clinical assessments. They will be advised that should there be a sustained decrease in either spirometry or pulse oximetry further clinical assessment is recommended.

Participants should continue to have all clinical appointments and medications as per their treating clinician’s recommendations. If there is a clinical decision to discontinue remote monitoring—either for safety/clinical reasons or due to patient choice this should be recorded in the electronic case report form (eCRF).

Usual care arm: These participants will have all clinical appointments and medications as per their treating clinicians’ recommendations.

All participants will be asked to complete health-related QoL questionnaires at 3 months (K-BILD, GAD-7), 6 months (K-BILD, GAD-7, EQ-5D-5L, PAM, modified MRC dyspnoea scale) and at 12 months which is the conclusion of the study.

All clinical observations, appointments, investigations and conversations which occur over the course of the following year will be recorded in the eCRF and analysed.

#### Outcomes

The primary outcome will be measured by comparing the number of clinical appointments where recent spirometry is available for review between the remote monitoring and usual care arms. Recent spirometry is defined as within 2 weeks of the clinical appointment. This will be measured at the first and last clinical appointments in the observation period.

The study hypothesis is that participants who have remote monitoring are more likely to have spirometry available for review at their clinic appointments and that the availability of spirometry is therefore dependent on the method of its collection.

Secondary endpoints of the study are as follows:

Service provision:

- Number of clinical reviews per patient.
- Proportion of clinical reviews which are scheduled, patient initiated and clinician initiated.
- Proportion of clinical reviews which are virtual versus face to face.
- Number of in-clinic lung function appointments/patient.
- Number of patients with chest CT scans.

Clinical:

- Proportion of patients with ≥10% decrease in forced vital capacity (FVC) (patient-recorded and hospital) of patient-recorded FVC.
- Change in FVC over time (patient-recorded and hospital).
- Number of patients starting antifibrotic medication, new immunosuppression and/or oxygen therapy.
- Patient-recorded dyspnoea score (mMRC score).
- HRQoL assessed by the EQ-5D-5L,^30^ K-BILD health status questionnaire^31^ and GAD-7.^32^

Adherence to study measurements:

- Adherence to study measurements in active arm.
- Proportion of patients recording measurements on ≥66% of weeks.
- Patient engagement and activation assessed by the 13-point PAM questionnaire.^33 34^

Health economic:

- Nursing/medical time reviewing data portal.
- Number of patient contacts with/by remote monitoring clinical team.
- Number of hospital admissions related to respiratory disease.

The study will be evaluated from a health economics perspective to compare NHS resource use in both arms.

#### Participant timeline

**Figure 1:**
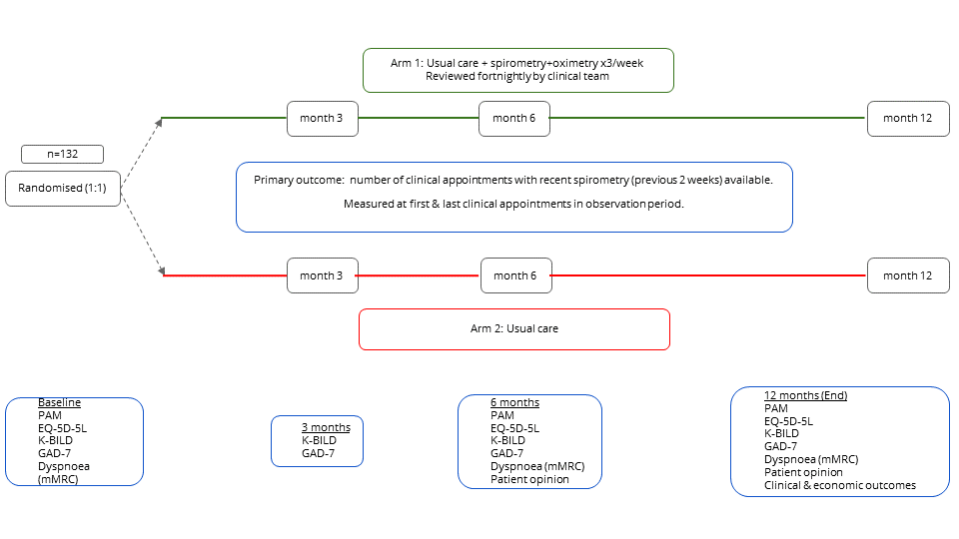
Diagram demonstrating the participant Timeline. EQ-5D-5L, EuroQol 5-Dimension 5-Level; GAD-7, Generalised Anxiety Disorder-7; K-BILD, Kings Brief Interstitial Lung Disease Questionnaire; mMRC, modified Medical Research Council; PAM, Patient Activation Measure.

The participant timeline is summarised in figure 1.

#### Sample size calculation

A power calculation was conducted using data from our clinical service at ICHT and data from the INMARK^18^ and INJUSTIS^17^ studies.

Retrospective analysis of our clinical service from 2019 reported spirometry was available at 79% of 131 appointments. (Data from 2019 were selected rather than more recent data as the authors were concerned that spirometry availability was disproportionately lower in 2020 and 2021 due to the impact of the COVID-19 pandemic).

Previous studies have shown high rates of adherence to daily and weekly home spirometry. Median adherence to weekly spirometry in the INMARK study was 96%.

The power calculation estimated that a sample of 108 participants would be large enough to detect a standardised effect size of 0.30 (Cohen’s w) between the active arm and usual care with 80% power. The rate of completion of the 12-month observation period in this study is estimated to be 82% (based on the completion rate of the 52-week INMARK study). Therefore, a total of 132 participants will be recruited to ensure that 108 complete the study.

#### Assignment of arms

Participants will be randomised using a random sequence, random block number randomisation schedule provided by an independent centralised electronic randomisation service (Sealed Envelope) Randomisation will be stratified by the ILD-GAP stage and whether the referring hospital is a secondary or tertiary care centre for ILD as this may affect the availability of formal lung function assessment within a lung function laboratory. ILD-GAP stage is calculated using the participants’ ILD diagnosis, age, sex and physiology and is associated with risk of mortality^35^ and has, therefore, been used here as a measure for severity of disease.

The study is not blinded to either participant or clinician.

#### Data collection and management

All clinical data will be collected on an eCRF provided by REDCap hosted by Imperial College Healthcare NHS Trust.^36 37^ Recruiting trusts are provided with login details on enrolment.

Participant questionnaires can be answered either on paper and transcribed onto REDCap or participants can be invited directly to answer questionnaires electronically through REDCap.

Data will be stored in REDCap during the duration of the project then downloaded and stored with the sponsor at conclusion of the project.

#### Data analysis plan

The objective of the primary analysis is to determine if the availability of spirometry within 2 weeks of the date of the first and last clinical review within the observation period is independent of the method by which it was obtained (ie, usual care with remote monitoring of spirometry or usual care alone). The primary endpoint variable is the number of outpatient clinical reviews for which spirometry data are available in the 2 weeks prior to the review. The primary endpoint will be assessed at the first and last outpatient clinical review in all patients who have ≥2 clinical reviews during the study observation period. The primary endpoint will be assessed by a χ^2^ test of independence.

All other endpoints will be summarised for descriptive statistical display.

The health economic analysis will be performed adopting the perspective of the NHS and Personal Social Services. NHS healthcare resource utilisation will be assessed using the data from the study and using standard unit cost data (Personal Social Services Research Unit), staff cost time and NHS tariffs. Effectiveness will be measured in terms of differences in quality-adjusted life-years in the two arms, using a linear approximation method, assuming the change in the utility over time is linear. Costs and effectiveness in both arms will be compared and the results presented in terms of incremental cost-effectiveness ratio.

#### Trial monitoring and oversight

The administrative information regarding the study is summarised in table 1.

**Table 1:** Summary of the administrative structure of the study

Study title	Comparison of clinical effectiveness and cost-effectiveness of a remote monitoring programme vs usual care in interstitial lung disease (REMOTE-ILD)	
Trial registration number Date registered	www.clinicaltrials.gov NCT05662124; 22/12/2022	
Protocol Version	v3.3 third July 2023	
Funding	The study is funded by NIHR Invention for Innovation (i4i) programme	
Protocol Contributors	Dr Sarah Barth Dr Colin Edwards Rebecca Borton Dan Beever Dr Wendy Adams Prof Gisli Jenkins Dr Elena Pizzo Dr Iain Stewart Dr Melissa Wickremasinghe	ICHT, ICL patientMpower Ltd patientMpower Ltd Action for Pulmonary Fibrosis Action for Pulmonary Fibrosis ICL UCL ICL ICHT
Name and contact information for the trial sponsor	Research Governance and Integrity Team, Joint research office, Room 221, Medical School Building, St Mary’s Campus, Norfolk Place, London W2 1 PG rgit@imperial.ac.uk	
Role of sponsor	The study sponsor has overseen the design of the study and will have oversight of the trial. The sponsor has ensured that the trial protocol, Patient Information Sheet (PIS), Informed Consent Form (ICF), GP letter and submitted supporting documents have been approved by the MHRA and a main Research Ethics Committee (REC), prior to any patient recruitment taking place. This study will be conducted in compliance with the protocol approved by the REC and according to GCP standards and UK Clinical Trials Regulation. Data ownership rights will lie with the institution. Our expectation is that after data analysis, information from this study will be widely disseminated in the medical and scientific community.	
Study start date	1^st^ March 2023	
Planned completion of study	31^st^ March 2025	

The study sponsor is Imperial College Healthcare NHS Trust. The sponsor can be contacted via rgit@imperial.ac.uk.

The study progress will be monitored fortnightly by a group with representatives of the primary research team at ICHT including the project manager, patientMpower and Action for Pulmonary Fibrosis (APF).

The trial sponsor has an annual audit programme and if the study is selected for audit it will be audited in accordance with the sponsor’s auditing procedures.

Any protocol modifications will be communicated following ethical approval to the responsible member at the participating sites to inform trial participants.

Informed consent will be obtained by the principal investigator and/or a nominated deputy as recorded on the sponsor’s delegation of responsibilities log. All participants will confirm their informed consent either in writing or electronically through the REDCap electronic consent form (online supplemental document 1).^38^

10.1136/bmjresp-2023-002067.supp1 Supplementary data

#### Safety reporting

No significant safety concerns are anticipated in relation to any measurements carried out as part of this study. All adverse events will be recorded and closely monitored until resolution, stabilisation or until it has been determined that the study intervention is not the cause. The chief investigator shall be informed immediately of any serious adverse events and shall determine seriousness and causality in conjunction with any treating medical practitioners.

### Patient and public involvement

Representatives of the APF charity are active members of the study group and have contributed to the design of the study and attend the fortnightly meetings to monitor the study’s progress. All patient-facing documentation has been reviewed together with patient representatives and user experience evaluation has been developed alongside patients both with and without prior experience of remote monitoring. APF will also support recruitment and dissemination of study findings to participants and the wider ILD community.

#### Confidentiality

All data will be handled in accordance with the Data Protection Act 2018.

Personally identifiable data (PID) is marked on the eCRF so that it can be excluded on data download for analysis. Subject initial and trial identification number (ID) will be used for identification. Further PID is only included for the use of the electronic consent form.

PID is required for the conduct of the remote monitoring arm in order to set up the remote monitoring and also in order to provide equipment to participants. A data processing agreement is in place to support this data sharing under the Data Protection Act.

CRFs have been designed by the CI and the final version approved by the sponsor and ethics committee. All documents will be stored safely in confidential conditions. On all study-specific documents, other than the signed consent form, the participant will be referred to by the study participant number, not by name.

All investigators will have access to the anonymised data set for the purpose of analysis only and it will be stored by the sponsor.

#### Dissemination

All data will be anonymised and grouped for presentation and publication. The results from this study will be publicised at regional and national conferences as well as being submitted for publication in open access peer-reviewed journals in accordance with UK Research Council policies. No participants will be identified in any publications that arise from this research.

## Conclusion

REMOTE-ILD is a randomised controlled trial of remote monitoring of home spirometry and pulse oximetry in patients with ILD which will compare clinical and health economic outcomes to that of usual clinical care over 12 months.

## Data Availability

Data sharing not applicable as no datasets generated and/or analysed for this study. Anonymised aggregate data will be shared on reasonable request as detailed in the ethics approval.
