# Supplementary material for: REMOTE-ILD study: Description of the protocol for a multicentre, 12-month randomised controlled trial to assess the clinical and cost-effectiveness of remote monitoring of spirometry and pulse oximetry in patients with interstitial lung disease
Source: BMJ Open Respir Res. 2024 Feb 28;11(1):e002067. doi: 10.1136/bmjresp-2023-002067 (PMC10910426; doi:10.1136/bmjresp-2023-002067)
Supplement: Supplementary data [file bmjresp-2023-002067supp001.pdf]

NHS
Imperial College Healthcare
NHS Trust

Consent Form

Full Title of Project: Comparison of health outcomes of a remote monitoring programme versus usual care in Interstitial Lung Disease

IRAS number: 301636
Name of Principal Investigator: Dr Melissa Wickremasinghe
Version 3.3 Date: 21st December 2023

Please initial to indicate your consent with the statements below.

Please initial box

1. I confirm that I have read and understand the patient information sheet dated … version … for the above study and have had the opportunity to ask questions which have been answered fully. ☐

2. I understand that my participation is voluntary and I am free to withdraw at any time, without giving any reason and without my medical care or legal rights being affected. ☐

3. I understand that sections of any of my medical notes may be looked at by responsible individuals from Imperial College NHS Trust or from regulatory authorities where it is relevant to my taking part in this research. ☐

4. I give permission for these individuals to access my records that are relevant to this research. ☐

5. I understand that relevant sections of my data collected in the study may be looked at by authorised individuals from Imperial College London, University College London and the research group at patientMpower Ltd. I give permission for these individuals to have access to these records and to collect, store, analyse and publish information obtained from my participation in this study for up to 5 years. I understand that my personal details will be kept confidential. ☐

6. I give consent for any information collected about me to be used to support other research in the future, including those outside of the EEA. yes ☐ no ☐

7. I understand that my name, postal address, telephone number and email address will be shared with patientMpower Ltd.

   This information will be retained until the analysis of the project is complete and will then be deleted. ☐

8. I understand that in order to conduct this study, the research team and the intervention providers may need to share personal information about me, such as my contact information and details of my health status with each other.

   I understand that this data will only be used for research purposes or to ensure I receive the best clinical care and I give permission for my personal information to be used in this way. ☐

9. I understand that information about me will be shared with Sealed Envelope Ltd and Insignia Health plc in order to carry out the study. I understand that I will not be able to be identified from this information. ☐

10. I consent to take part in the above study. ☐

Barth S, et al. BMJ Open Respir Res 2024; 11:e002067. doi: 10.1136/bmjresp-2023-002067

Imperial College Healthcare NHS Trust

11. I give consent to my general practitioner (GP) being informed that I am taking part in this study. ☐

12. I give consent to being contacted to potentially taking part in other research studies. yes ☐ no ☐

13. I give consent to potentially being contacted up to 5 years in the future with regards to this study. yes ☐ no ☐

14. I give consent for any data I enter into the patientMpower app for up to 5 years after the study is completed to be analysed and published. ☐

Name of Subject	Signature	Date
Name of Person taking consent	Signature	Date

1 copy for subject; 1 copy for Principal Investigator; 1copy to be kept with hospital notes

Barth S, *et al. BMJ Open Respir Res* 2024; 11:e002067. doi: 10.1136/bmjresp-2023-002067
